# Effects of Noise Exposure on the Vestibular System: A Systematic Review

**DOI:** 10.3389/fneur.2020.593919

**Published:** 2020-11-25

**Authors:** Courtney Elaine Stewart, Avril Genene Holt, Richard A. Altschuler, Anthony Thomas Cacace, Courtney D. Hall, Owen D. Murnane, W. Michael King, Faith W. Akin

**Affiliations:** ^1^University of Michigan Department of Otolaryngology/Head-Neck Surgery, Kresge Hearing Research Institute, Ann Arbor, MI, United States; ^2^VA Ann Arbor Healthcare System, Research Service, Ann Arbor, MI, United States; ^3^Department of Ophthalmology Visual and Anatomical Sciences, Wayne State University School of Medicine, Detroit, MI, United States; ^4^John D. Dingell VA Medical Center, Molecular Anatomy of Central Sensory Systems Laboratory, Research Service, Detroit, MI, United States; ^5^Department of Communication Sciences and Disorders, Wayne State University, Detroit, MI, United States; ^6^Department of Rehabilitative Sciences, Doctor of Physical Therapy Program, East Tennessee State University, Johnson City, TN, United States; ^7^Gait and Balance Research Laboratory, James H. Quillen VA Medical Center, Mountain Home, TN, United States; ^8^Department of Audiology and Speech-Language Pathology, East Tennessee State University, Johnson City, TN, United States; ^9^Vestibular Research Laboratory, James H. Quillen VA Medical Center, Mountain Home, TN, United States

**Keywords:** vestibular system, noise-induced vestibular loss, saccule and utricle, semicircular canals, impulse noise, continuous noise, vestibular nuclear complex

## Abstract

Despite our understanding of the impact of noise-induced damage to the auditory system, much less is known about the impact of noise exposure on the vestibular system. In this article, we review the anatomical, physiological, and functional evidence for noise-induced damage to peripheral and central vestibular structures. Morphological studies in several animal models have demonstrated cellular damage throughout the peripheral vestibular system and particularly in the otolith organs; however, there is a paucity of data on the effect of noise exposure on human vestibular end organs. Physiological studies have corroborated morphological studies by demonstrating disruption across vestibular pathways with otolith-mediated pathways impacted more than semicircular canal-mediated pathways. Similar to the temporary threshold shifts observed in the auditory system, physiological studies in animals have suggested a capacity for recovery following noise-induced vestibular damage. Human studies have demonstrated that diminished sacculo-collic responses are related to the severity of noise-induced hearing loss, and dose-dependent vestibular deficits following noise exposure have been corroborated in animal models. Further work is needed to better understand the physiological and functional consequences of noise-induced vestibular impairment in animals and humans.

## Introduction

**Figure 1:**
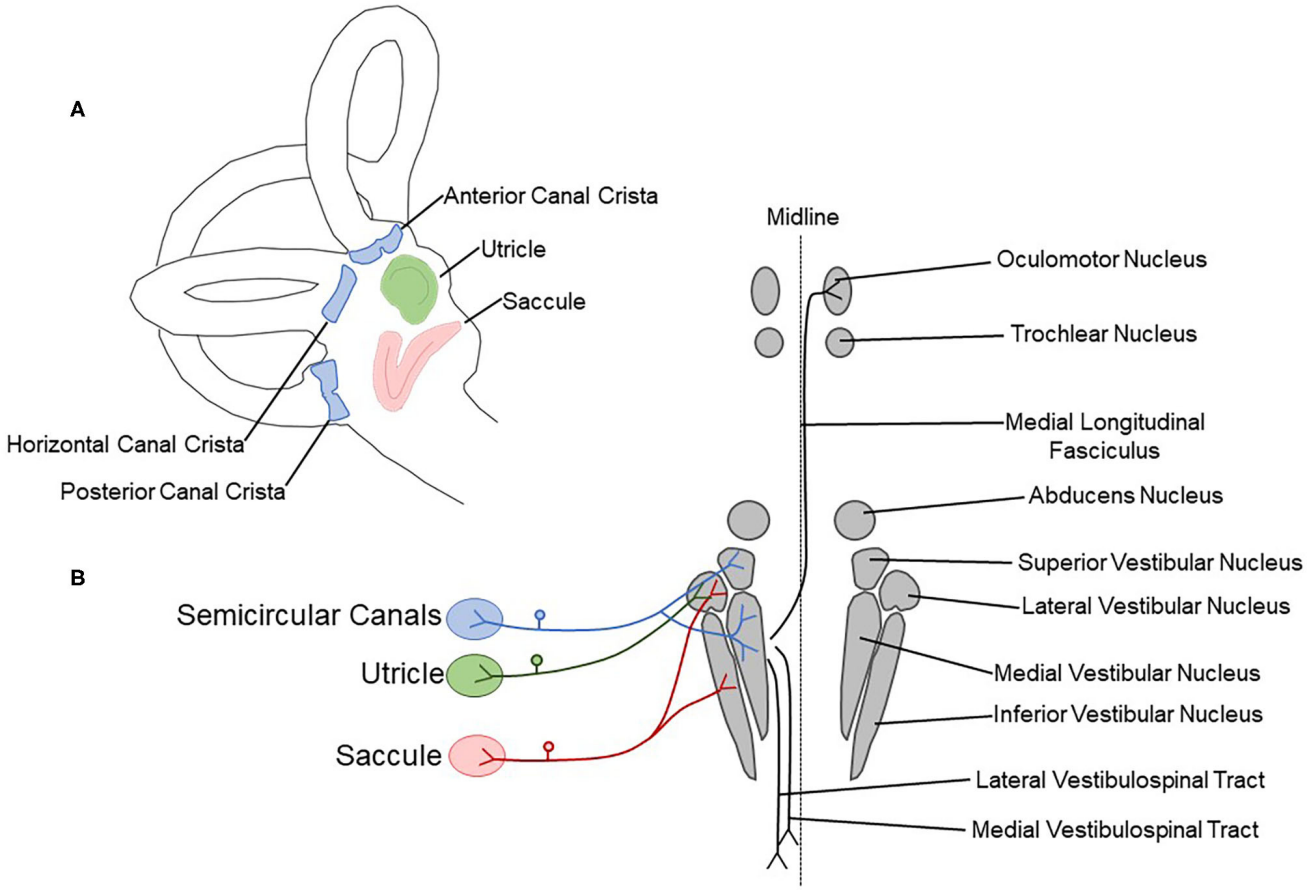
Schematic drawing showing peripheral vestibular sensory organs and central pathways. **(A)** The vestibular labyrinth contains three semicircular canal cristae (anterior, horizontal, and posterior; blue) and two otolith organs (saccule, red; utricle, green). **(B)** The semicircular canal cristae, utricle, and saccule are innervated by the vestibulocochlear nerve (CN VIII). Afferent fibers terminate in the vestibular nuclear complex, containing the superior, lateral, medial, and inferior vestibular nuclei. The medial longitudinal fasciculus, lateral vestibulospinal tract, and medial vestibulospinal tract are critical central components of vestibular reflex pathways.

It is well-established that noise overstimulation has the potential to cause temporary or permanent damage to sensory cells in the cochlea and the afferent neurons innervating them, resulting in temporary or permanent loss of hearing [for review see: (1, 2)]. Less known and considerably less understood are the effects of noise on vestibular and balance function. Similar to the cochlea, the vestibular sensory end organs are housed within the temporal bone and membranous labyrinth of the inner ear. Hair cells, the sensory cells of the inner ear, share similar morphology in the vestibular end organs and in the organ of Corti; they both transduce displacement of hair-bundles into neural activity through the shared vestibulocochlear nerve (CN VIII). Five peripheral vestibular end organs (three semicircular canal cristae and two otolith organs) provide sensory input to vestibular nuclei as well as the vestibular cerebellum and contribute to vestibulo-ocular and vestibulo-spinal reflexes (VOR and VSR; Figure 1). Although a primary role of the mammalian vestibular system is to maintain gaze and postural stability, neurophysiological studies demonstrate that, like the cochlea, the vestibular end organs, and the saccule and utricle (otolith organs) in particular, are sensitive to sound [e.g., (3–5) for reviews see (6, 7)]. Large diameter afferents with calyceal terminations are characterized by phase-locking and an irregularly discharging firing rate (8–10), high sensitivity to linear forces (11), and increased firing in response to air-conducted sound or bone-conducted vibration (3, 4, 12). The properties of irregular vestibular afferents have been described in detail [(13); for reviews see (6, 14)]. Their physiological properties and sound-sensitivity put these afferents at greater risk for noise-induced damage. Specifically, since irregular vestibular afferents can be activated by sound, it follows that this population may be over-stimulated by sound, and therefore susceptible to noise-induced damage. Noise exposures can be grouped into one of two types—impulse or continuous noise. Continuous noise occurs over an extended period of time, whereas impulse noise occurs rapidly, and generally at a considerably higher sound pressure level (SPL). Both types of noise exposures will be explored in this review with a description of the anatomical, neurophysiological, and functional evidence for noise-induced damage to the vestibular system.

## Anatomical Evidence for Noise-Induced Damage to the Vestibular Periphery

### Continuous Noise Exposure

Similar to the auditory system, animal models of continuous noise exposure have revealed that damage to the vestibular periphery is dependent on characteristics of the noise, including: duration, frequency, level, and time course. The duration of noise exposure varies across experiments ranging from <1 h to more than 1 day and likely contributes to the level of tissue damage observed across studies. In an early study, Mangabeira-Albernaz et al. (15) used a wide range of frequencies (170–50,000 Hz) and durations (5–160 min) at high SPLs (118–133 dB), and then allowed 0–133 days of recovery before tissue collection. Across all of the tissue analyzed, saccular collapse was observed in approximately one third of the samples and utricular rupture was observed in approximately one third of the samples. Saccular rupture was identified in ~25% of the samples, and utricular collapse was least commonly observed, in ~15% of samples. When this damage is categorized by frequency, saccular rupture was most prevalent with 0.5–2 kHz noise exposure (142–150 dB SPL, 1–4 min), and saccular collapse was most prevalent with 4 kHz noise exposure (150–163 dB SPL, 1–2 min). Utricular rupture was most prevalent with 1–4 kHz, and 40–50 kHz (142–163 dB SPL, 1–4 min and 140–144.5 dB, 2–4 min, respectively), and utricular collapse did not appear to occur at a specific frequency. Interestingly, rupture of the saccule was not changed with post-noise exposure recovery time, but rupture of the utricle became less common as post-noise exposure recovery time increased. Conversely, utricular collapse was not impacted by post-noise exposure recovery time, but saccular collapse was more common as post-noise exposure recovery time increased. This study laid the groundwork for more recent work and provided early evidence of noise-induced damage to the vestibular periphery.

Hsu et al. (16) demonstrated the impact of duration of noise exposure on tissue damage by delivering a broadband noise of identical intensity (115 dB SPL) to guinea pigs for either 30 min or 40 h. Assessment of general morphology using light and electron microscopy showed no signs of saccular disruption 1 week after noise exposure with the short term 30-min exposure. The long-term 40-h exposure resulted in otolithic membrane disruption, as well as atrophy and vacuolization in type I saccular hair cells. There was little damage to type II hair cells or supporting cells and the vestibular nerve was intact 1 month after noise exposure (16).

**Figure 2:**
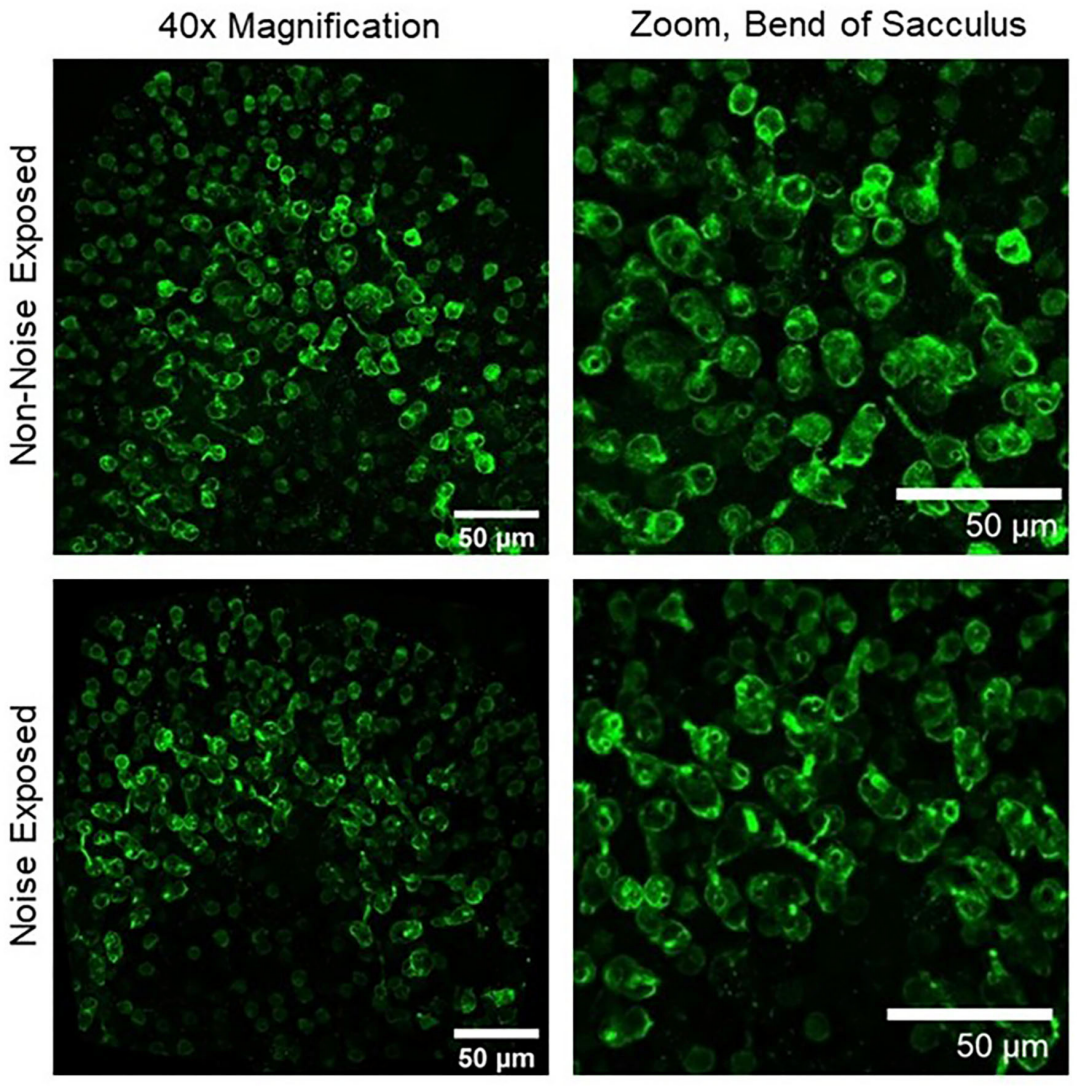
Upper left, 40× magnification image of a non-noise exposed sacculus labeled with calretinin, a marker of calyx-only afferent terminals. Upper right, zoomed image of the bend of the sacculus showing numerous well-labeled calyces in non-noise-exposed tissue. Lower left, 40× magnification image of a noise-exposed sacculus. Lower right, zoomed image of the bend of the sacculus showing a reduction in the number of calretinin-labeled calyces 28 days after noise exposure (18).

**Figure 3:**
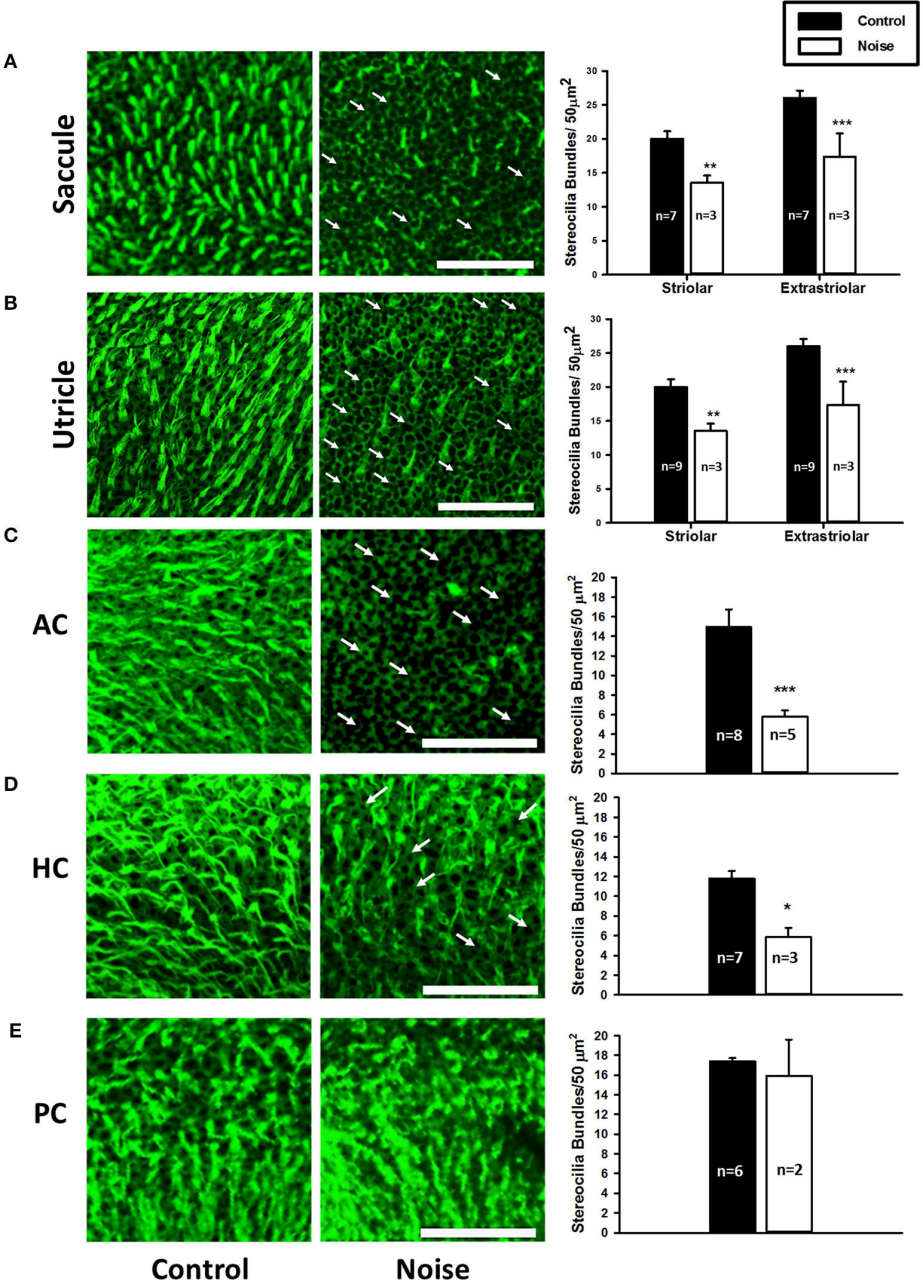
**(A,B)** Representative images of the saccular macula and the utricular macula stained with phalloidin from control (left panels) and noise-exposed rats (right panels). Arrows indicate intact cuticular plates with missing stereocilia bundles. Scale bar is 50 μm. Noise exposure decreases stereocilia bundle density in both the striolar and extra-striolar regions of the saccules and the utricles (**P < 0.01; ***P < 0.0005). **(C–E)** Representative images of the anterior (AC), horizontal (HC), and posterior (PC) semicircular canal cristae stained with phalloidin from control (left panels) and noise-exposed rats (right panels). Arrows indicate missing stereocilia bundles. Scale bar is 50 μm. Noise exposure decreases stereocilia bundle density in the AC and HC, but not the PC (*P < 0.05; ***P < 0.0005). Adapted from (19).

The time course of noise exposure also influences the potential for peripheral vestibular damage. Akdogan et al. (17) compared vestibular changes in guinea pigs exposed to an intense (120 dB SPL) 6-h 4 kHz octave band (continuous) noise vs. a group exposed to intermittent noise (1-h exposure followed by a 1-h break, alternating for 12 h). Damage was identified in the continuous, but not the intermittent noise exposure group. This damage included large vacuoles and enlarged mitochondria with crystolysis in epithelial cells from saccular maculae and apoptosis of non-sensory cells (stromal cells and osteocytes). This study suggests that intermittent noise exposure is less damaging to the vestibular system than continuous noise exposure. In a similar study, rats were exposed to a continuous 6-h intense (120 dB SPL) 1.5 kHz 3-octave band noise. Figure 2 shows significant decrease in calretinin immunolabeled calyceal endings observed in rat saccular maculae following a 28-day recovery period; however, hair cell loss was not observed in this study (18). Following a 3-h 116 dB SPL broadband noise exposure, damage to stereocilia bundles without qualitative observation of hair cell loss (absence of scarring where hair-bundles were missing in sensory epithelia) was significant through most of the vestibular labyrinth (saccule, utricle, and anterior and horizontal semicircular canals), with the greatest effect observed in the otolith organs in tissue collected 7 days after noise exposure [(19); Figure 3].

The sound levels used to study the effects of continuous noise on the vestibular periphery have ranged from 70 to 150 dB SPL with even higher levels used in impulse noise exposure paradigms. Exposure to lower sound levels over a long time period can produce signs of peripheral vestibular injury (20), whereas higher sound levels can produce damaging effects within minutes (21). Tamura et al. (20) observed a reduction in the number of vestibular hair cells and increased oxidative stress in mice exposed to a relatively low sound level (70 dB SPL) for 1 month. In contrast, a 20-min exposure to a much higher sound level (136 to 150 dB SPL) band-limited noise produced saccular collapse, destruction of otolithic membrane, and detached macular sensory cells (21). It should be noted, however, that in this study the non-saccular vestibular end organs were not affected by the noise exposure.

Evidence for frequency-dependent noise-induced damage has also been described. Tamura et al. (20) examined otolith organs collected from mice that were chronically exposed to low-intensity (70 dB SPL), low-frequency (0.1 kHz) noise. They identified fewer vestibular hair cells and elevated markers of oxidative stress including D-beta-aspartic acid and oxidized phospholipids compared to control animals. Interestingly, damage was not observed in animals exposed to the same duration and level of high-frequency (16 kHz) noise. These results are consistent with the finding that vestibular afferents are most sensitive to low-frequency sound stimulation [e.g., (3)].

**Figure 4:**
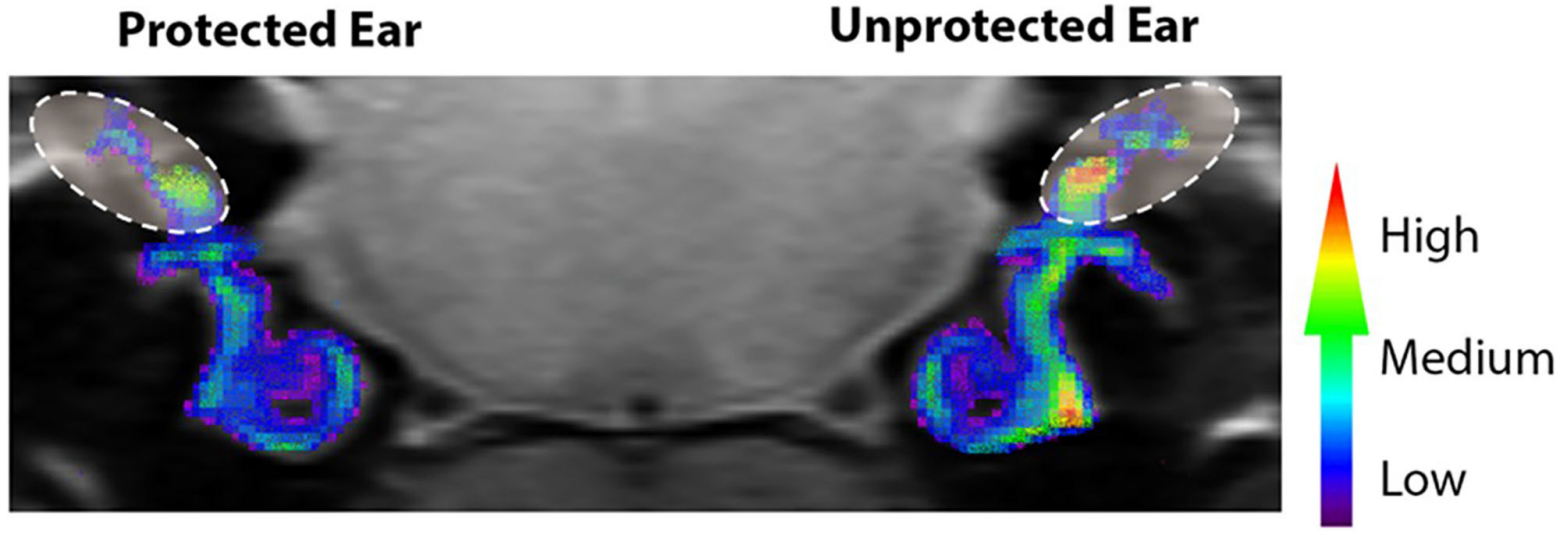
Noise-evoked inner ear oxidative stress measured *in vivo* with QUEST MRI. R1 (1/T1) maps were generated and used to compare protected (plugged ear) and unprotected vestibular end organs and cochleae. Scale bar is a graded colorimetric representation of R1 values. High values are represented as a gradient from red to yellow and low values are represented from blue to purple. Vestibular regions–area within ovals with dashed lines. The R1 values were collected *in vivo* from 400 μm MRI scans. Scans through the cochlear and vestibular ducts were sampled and analyzed from caudal to rostral. Therefore, the coronal image in Figure 4, provides views of the regions of interest from caudal to rostral (e.g., the right cochlea is viewed on the right side of the image). Adapted from (22).

The studies reviewed in this section have outlined the consequences of continuous noise exposure and mechanisms underlying damage to the vestibular periphery. Although noise-induced vestibular damage is attributed to excitotoxicity (especially when type I hair cells and calyceal afferents are preferentially impacted) and to direct mechanical trauma, ischemia and free-radical production also contribute to noise-induced damage observed in the vestibular periphery. Using quench-assisted magnetic resonance imaging (QUEST MRI) to measure excessive free radical production *in vivo*, Kühl et al. (22) identified noise-induced free radical production not only in the cochlea, but also in the vestibular aspect of the inner ear of rats exposed to 118 dB SPL, 10 kHz-centered 1/3 octave band noise for 4 h. When compared to normal hearing controls and ears protected from noise exposure by silicone elastomer, the unprotected cochlea of noise exposed rats exhibited elevated MRI R1 values. These increased MRI R1 values were “quenched” by anti-oxidant treatment, indicating the presence of noise induced free radicals. Measurement of MRI R1 values *in vivo* within vestibular related regions of the inner ear in these same animals suggest increased free radical levels after noise exposure (Figure 4). Other studies have used post-life measures of noise-induced free radical production. Fetoni et al. (23) exposed guinea pigs to a 6 kHz pure tone at 120 dB SPL for 1 h and identified hair cell loss, a large, progressive increase in vascular endothelial growth factor (VEGF), and a small increase in 4-hydroxynonenal (4-HNE). 4-HNE is a product of lipid peroxidation and used as a marker of oxidative stress. VEGF is primarily viewed as an angiogenic factor; it has been suggested that it is also protective against apoptosis (24, 25) and is upregulated in noise-induced hearing loss (26–28). It is possible that VEGF is induced by ischemia (24) and related to the production of reactive oxygen species (29). Tamura et al. (20) exposed mice to a continuous 0.1 kHz noise at 70 dB SPL for 1 month. After noise exposure, the inner ears were paraffin-embedded and sectioned. In sections of the vestibule that contained the otolith organs, hair cell loss and elevated levels of oxidative stress were observed. Oxidative stress was determined as elevated presence of oxidized phospholipids and D-beta-aspartic acid. Both studies suggest that noise exposure can lead to production of free-radicals; however, differences in the identification of free-radicals by Tamura et al. (20) and Fetoni et al. (23) are likely due to differences in the animal model, the selection of antibody targets, and the duration of post-noise recovery prior to tissue analyses. It is also possible that a long duration or a low frequency noise exposure produces the greatest damage, a finding that is relevant to environmental health and workplace safety.

In summary, these studies suggest that both brief exposure to an elevated sound level and sustained exposure to low-frequency continuous sound at more moderate levels can have a damaging impact on the vestibular periphery. Comparisons across studies are difficult due to differences in frequency range, duration, sound level, time course, and even animal models; however, it is clear that vestibular damage is measurable following noise exposure. Animal models have revealed cellular and anatomical changes to the vestibular periphery associated with noise overstimulation. Continuous noise-induced damage has been identified with markers of oxidative stress and ischemia; fewer calyceal terminals, loss of stereocilia bundles, hair cell loss, and, with sufficient sound pressure, a complete collapse of the saccule, and destruction of the otoconial matrix overlaying this structure. Most of the research on the effects of continuous noise on the vestibular periphery has examined the impact on the otolith organs, and the saccule is likely the most susceptible to noise-induced damage due to the anatomical proximity of the saccule to the stapes footplate (30) [e.g., (17, 19, 21)]. In contrast, fewer studies have examined noise-induced damage to the semicircular canals (19).

### Impulse Noise Exposure

To our knowledge, there is no report that has examined the vestibular aspect of the human temporal bone after continuous or impulse noise exposure. Kerr and Byrne (31), however, examined temporal bones of two victims killed in a Northern Ireland restaurant bombing and their histological examination revealed rupture of the saccule, utricle and basilar membranes following a close-proximity blast.

In guinea pigs exposed to impulse noise from 90 to 300 rifle shots with a peak level of 158 dB SPL at 1.1 kHz, Ylikoski (32) found that the most severe damage occurred in ampullary cristae and the cochlea. This damage was characterized as a separation of sensory epithelium from underlying connective tissue and damage to sensory cells. With this noise exposure condition, epithelial separation occurred less frequently and was less severe in utricular and saccular maculae than in cristae (32). In contrast, Lien and Dickman (33) exposed mice to 63 kPa peak blast waves and observed stereocilia bundle loss in the utricular maculae and horizontal semicircular canal cristae (the sacculus and vertical semicircular canals were not measured), suggesting a broader effect of blast exposure than is seen in other noise exposure paradigms.

Kumagami (34) reported that moderate to extensive endolymphatic hydrops occurred 1 year after exposure to firecracker explosion in Albino guinea pigs. According to the author, from 6 months to 1-year post-exposure, the vestibule and semicircular canals showed slight to moderate endolymphatic hydrops without overt damage to the sensory maculae contained within these structures. In this temporal trajectory, three prominent post-traumatic events occurred: (1) after 4 months, degeneration of the endolymphatic sac was observed, (2) endolymphatic hydrops developed after disappearance of the Preyer reflex in ~50% of the animals studied, and (3) damage to cochlear hair cells preceded degeneration of epithelial cells in the endolymphatic sac. In summary, these studies suggest that impulse noise may have a broad impact, damaging the ampullary cristae, otolith organ maculae, and endolymphatic sac; however, literature on the impact of impulse noise on tissue damage in the vestibular periphery is limited.

## Anatomical Evidence for Noise-Induced Damage to Central Vestibular Pathways

There is some anatomical evidence for central vestibular pathway damage following exposure to continuous and impulse noises. A gas chromatography mass spectrometry (GC/MS)-based metabolomics platform has been used to show changes in neurotransmitter-related metabolites after exposing rats unilaterally for 1 h to a 16 kHz 110 dB SPL tone (35). After 6 months, increases in glutamic acid were found in both the vestibular nuclear complex (VNC) and the cerebellum. There were also significant increases in the relative abundance of cysteine, urea, and inosine in the VNC while glycine, 3-hydroxybutyrate, and myo-inositol concentrations were elevated in the cerebellum. These chronic changes in metabolites are suggestive of changes in neuronal activity and the balance between excitation and inhibition (i.e., increased endocytosis).

Kaur et al. (36) evaluated the cerebellar cortex of rats in response to blast exposure. When examined 4 to 7 days after the blast exposure, ultrastructural analysis using electron microscopy revealed neurons with darkened dendrites (dark appearance of cytoplasm within dendritic processes). Darkened dendrites can indicate that neurons are in an atrophic state due to trauma and have been reported following axotomy and exposure to neurotoxins (37, 38). Additionally, microglia at this same time point exhibited morphological changes and proliferation, suggesting a massive inflammatory response mediated by microglia. Microglia were observed near and sometimes even wrapping around some of the darkened dendritic processes. At 21–28 days after the blast, however, no darkened dendrites were observed, and the morphological characteristics and numbers of microglia had returned to pre-blast levels. The data suggest that these proliferating microglia removed, at least a portion of, the affected dendrites. The authors postulated that acute changes in affected neurons and activation of microglia may have resulted in a prolonged atrophic state and/or release of factors that caused other chronic effects (e.g., changes in neuronal activity, increased endocytosis).

Although some morphological changes were acute as reported by Kaur et al. (36), studies in other animal models showed persistent effects. The impact of mild repetitive blast (3 blasts 20–30 min apart ranging from 15–19 psi = 107–133 kPa) on brain microstructure and volume was examined using magnetic resonance imaging (MRI) in Sprague Dawley rats, at two time points (7 and 90 days) after exposure (39). At 90 days post-trauma, localized reductions in volume were observed in the cerebellum and the VNC. Microstructural changes in the cerebellum were observed at 7 days and persisted through the 90-day time point. The specific details surrounding the morphological changes were not reported. Although ipsilateral vs. contralateral damage was discussed, neither damage of specific subnuclei nor localization within subnuclei were described (i.e., rostro-caudal, dorso-ventral, or medio-lateral). Another question is whether particular neuronal phenotypes were disproportionately affected (e.g., excitatory vs. inhibitory). Other studies have addressed the differential impact of continuous noise on neurons within vestibular nuclei. Specifically, in Barker et al. (40), neurons in the lateral vestibular nucleus (LVN) of the rat project to the dorsal cochlear nucleus (DCN). Combining tract tracing and immunohistochemistry 5 days after a noise trauma (15 kHz, 110 dB SPL for 6 h), synaptic terminals originating from LVN neurons were shown to be more numerous in the DCN when compared to sham noise-exposed control animals. They further determined that the synaptic terminals were glutamatergic, immunolabeling for vGLUT2 (vesicular glutamate transporter 2), a protein responsible for loading glutamate into synaptic vesicles. Others have postulated that neuropathic pain and inflammatory processes induced by noise may underlie the increased excitatory vGLUT2 neurite outgrowth from the LVN into the cochlear nucleus [e.g., (41)]. These new synaptic terminals could be evidence of cross-modal plasticity and contribute to an increase in spontaneous neuronal activity that is sometimes observed following loud noise exposure and could be associated with the perception of tinnitus and/or hyperacusis.

## Electrophysiological Evidence for Noise-Induced Damage to the Vestibular System

**Figure 5:**
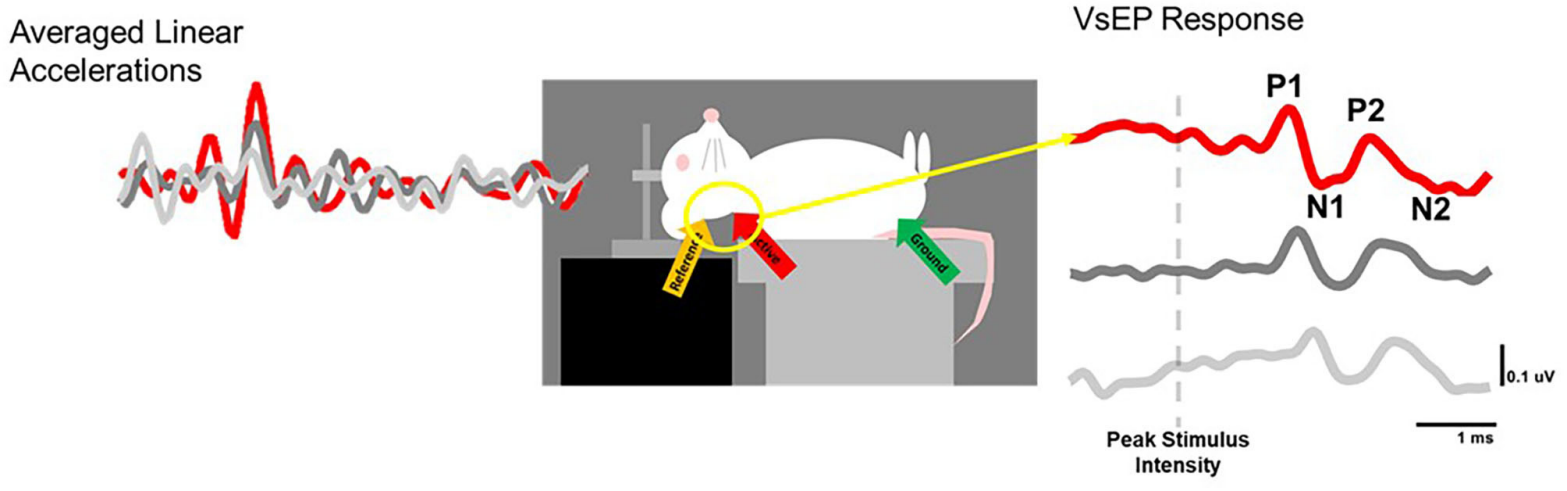
VsEP recording set-up. The skull of an anesthetized rat is fixed to the arm of an electrodynamic transducer (ET-132-203, Labworks, Inc.) and linear head-jerks (left) are delivered in the naso-occipital plane to elicit responses (right) with characterisitic P1N1 and P2N2 waveforms that can be measured before and after noise exposure to track vestibular loss and recovery over time.

Neurophysiological studies have focused primarily on otolith organ pathways in examination of the impact of noise exposure on vestibular function. The majority of animal studies have used the vestibular short latency evoked potential (VsEP) to study changes in central and peripheral activity after noise exposure [(18, 42–45) for review of VsEPs see: (46)]. VsEPs have predominantly been recorded from experimental animals in response to brief linear acceleration impulses applied to the skull using an electrodynamic shaker that is bolted or clamped to the animal's skull [Figure 5; e.g., (47)]. VsEPs remain intact following cochlear extirpation whereas the response is abolished following damage to the vestibule or eighth nerve or the administration of neural blocking agents [e.g., (48)]. Further, the use of air conduction masking does not eliminate the VsEP response (48, 49). For a review of the vestibular specificity of the VsEP, see Brown et al. (50). The VsEP reflects the synchronous compound field potential of peripheral and/or central vestibular neurons in response to the onset of head/body motion (jerk). The VsEP is well-validated and used to evaluate the effects of noise exposure on irregular otolithic afferents. It is known that irregular afferents that contribute to central vestibular reflex pathways are sound sensitive [e.g., (3); for review see (6)]. Furthermore, it has been demonstrated that damage to this population of afferents (Figure 2; calretinin-positive calyx-only afferent terminals) is associated with loss or reduction of the VsEP response (18, 45). In summary, the VsEP is an appealing metric to evaluate the vestibular consequences of noise exposure in animal models; however, due to challenges in recording VsEPs in humans, the impact of noise exposure on the human VsEP has not been examined.

**Figure 6:**
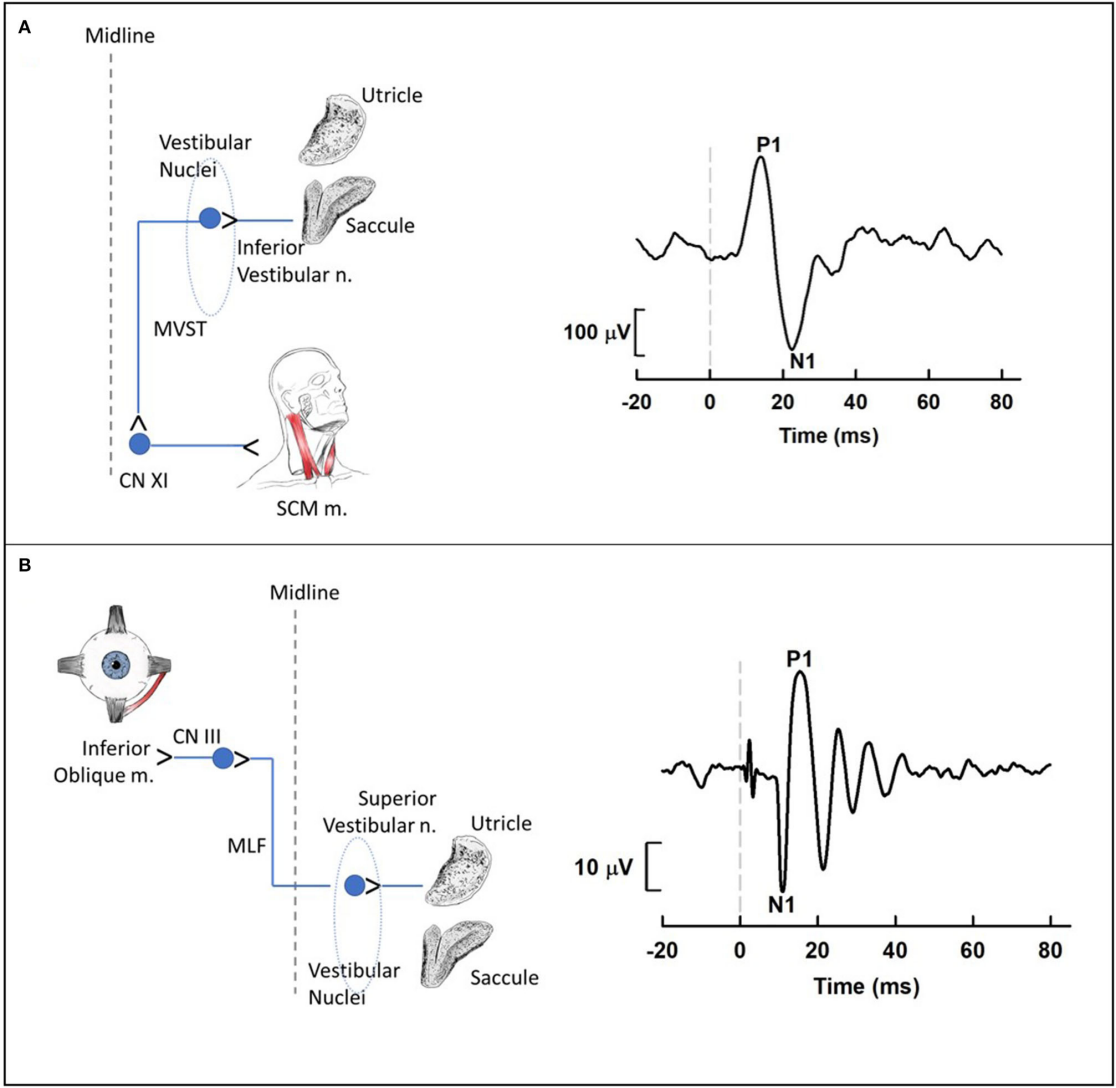
Cervical **(A)** and ocular **(B)** vestibular-evoked myogenic potential (VEMP) pathways and waveforms. **(A)** Cervical VEMPs (cVEMPs) are mediated by an ipsilateral reflex pathway originating in the saccule and projecting to motoneurons of the sternocleidomastoid muscle (SCM m.) via the inferior vestibular nerve, vestibular nuclei and descending medial vestibulospinal tract. The cVEMP waveform was obtained using air conduction 500-Hz tone bursts (95 dB nHL) during activation of the SCM m. with a lateral head turn. **(B)** Ocular VEMPs (oVEMPs) are mediated by a contralateral reflex pathway originating in the utricle and projecting to motoneurons of the inferior oblique muscle via the superior vestibular nerve, vestibular nuclei, medial longitudinal fasciculus, and the oculomotor nucleus. The oVEMP waveform was obtained using bone conduction 500-Hz tone bursts (Brüel & Kjær Model 4,810 mini-shaker applied to the midline forehead; 145 dB peak force level) during upward gaze. For each waveform, the dashed vertical line at 0 ms indicates stimulus onset. Medial Vestibulospinal Tract, MVST; Accessory Nerve, CNXI; Sternocleidomastoid, SCM; Oculomotor Nerve, CNIII; Medial Longitudinal Fasciculus, MLF.

To examine the impact of noise on the human vestibular system, most laboratories have used vestibular evoked myogenic potentials (VEMPs; Figure 6). VEMPs are short-latency myogenic potentials arising from vestibular afferents that are responsive to air-conducted sound or bone-conducted vibration (51). Cervical VEMPs (cVEMPs; Figure 6A), a measure of the sacculo-collic pathway, are recorded from surface electrodes over the sternocleidomastoid muscle. Ocular VEMPs (oVEMPs; Figure 6B), a measure of utricular/superior vestibular nerve function, are recorded from surface electrodes over the inferior oblique extraocular muscle. cVEMPs are mediated by an ipsilateral reflex pathway originating in the saccule and projecting to motoneurons of the sternocleidomastoid muscle via the inferior vestibular nerve, vestibular nuclei and descending medial vestibulospinal tract [for review, see (6)]. oVEMPs are mediated by a contralateral reflex pathway originating in the utricle and projecting to motoneurons of the inferior oblique muscle via the superior vestibular nerve, vestibular nuclei, medial longitudinal fasciculus, and the oculomotor nucleus [for review, see (52)].

### Otolith Organ Pathways

#### Vestibular Short-Latency Evoked Potentials (VsEPs)

**Figure 7:**
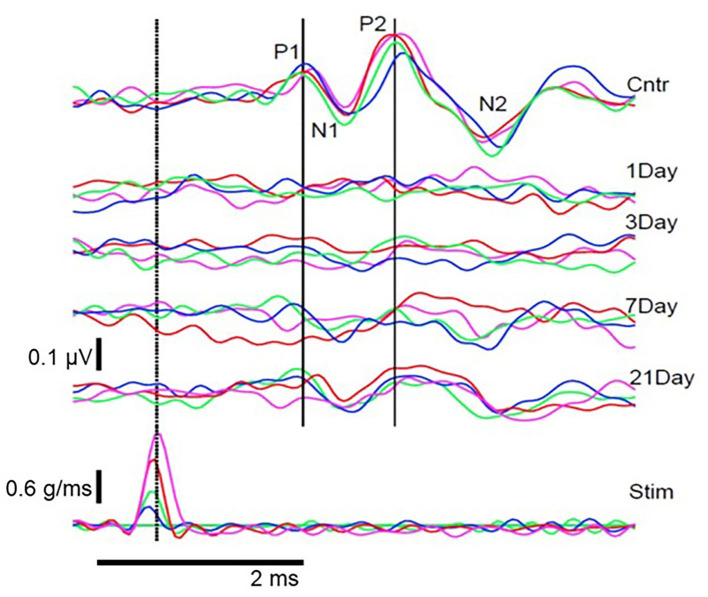
Pre- and post-noise exposure VsEP waveforms for 1 animal at 4 stimulus intensities. **(Bottom row)** stimulus waveforms at 4 intensities (blue, 0.2 g/ms; green, 0.4 g/ms; red, 0.7 g/ms; magenta, 1.2 g/ms). **(Top)** Pre-exposure (Cntr) and post-exposure (Day 1–21) VsEP waveforms for each stimulus intensity and the identified P1N1 and P2N2 components. VsEP is abolished immediately after noise exposure and partially recovers 3–21 days after exposure. Dotted vertical line marks peak stimulus intensity and was used as the reference (0 ms) to calculate latency. Originally published in (45).

**Figure 8:**
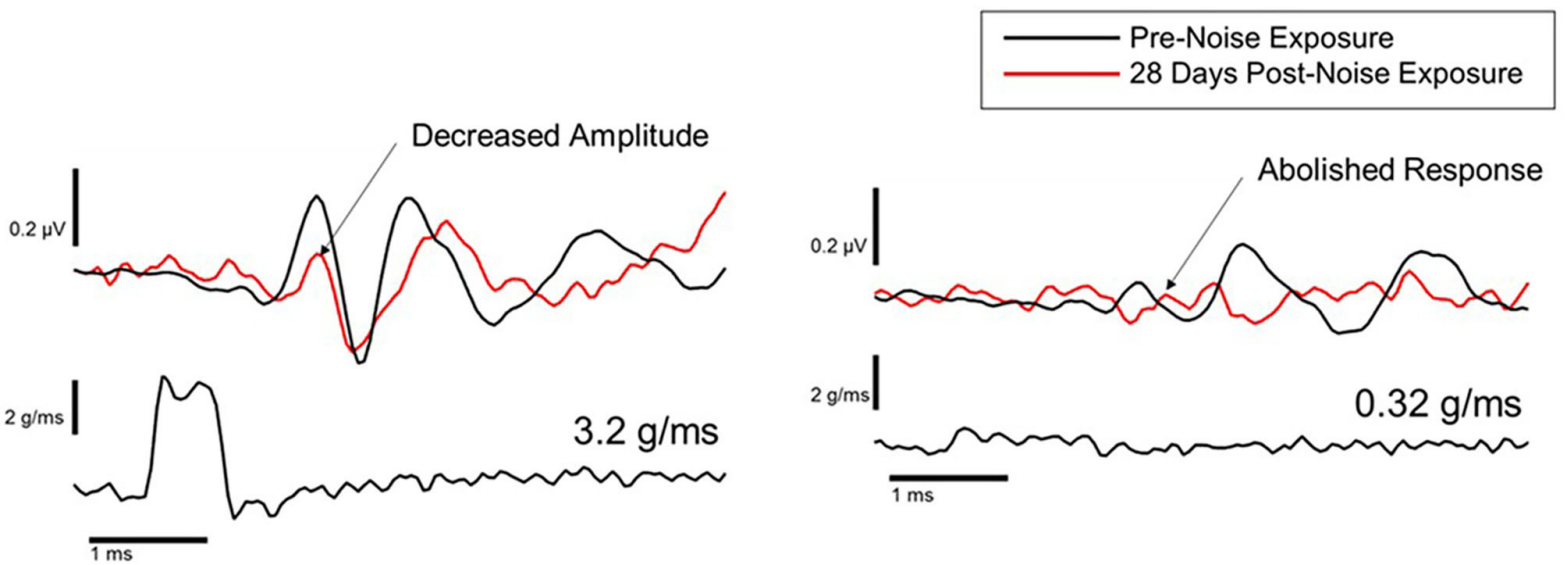
Representative VsEP waveforms in response to a 3.2 g/ms stimulus (left), and a 0.32 g/ms stimulus (right), at baseline (black traces) and 28 days after noise exposure (red traces) (18).

Animal studies using VsEPs have demonstrated changes in VsEP characteristics following noise exposure. Perez et al. (44) observed a reduction in VsEP amplitude elicited by a ~3 g/ms head-jerk in rats following exposure to impulse noise (10 gunshots at ~160 dB SPL). Six weeks after noise exposure, the VsEP amplitude recovered but the latency did not, suggesting an incomplete recovery. Similarly, Stewart et al. (45) reported a reduction in VsEP amplitude in rats exposed to high-intensity (120 dB SPL) low-frequency (0.5–4 kHz) continuous noise for 6 h. Unlike the Perez et al. (44) study, the initial reduction in VsEP amplitude using head-jerk stimuli up to 1.2 g/ms did not recover 3 weeks post-noise exposure (Figure 7). In a follow-up study that used the same noise exposure paradigm, larger head jerk stimuli were used to elicit VsEP responses and track recovery for 28 days post-noise exposure (18). Even with larger head-jerk stimuli, the post-noise exposure response amplitudes were severely attenuated and exhibited longer latencies than those obtained from the non-noise exposed animals. In fact, these deficits showed minimal recovery 28 days after noise exposure [(18); Figure 8]. It is likely that differences between the results of Perez et al. (44) and Stewart et al. (18, 45) were related, at least in part, to differences in the level and duration of the noise exposure paradigms. In the Perez et al. (44) study, the impulses delivered to the rats were 40 dB SPL greater than in the continuous noise paradigm used by Stewart et al. (18, 45). However, the effect of continuous noise delivered over 6 h was considerably more severe and more persistent. In contrast to the work of Stewart et al. (18, 45) and Perez et al. (44), two studies reported that continuous 113 dB SPL white noise did not induce a deficit in VsEP responses in intact animals (42, 43). No significant changes were observed in VsEP amplitude or latency after a 1-h exposure or after 3 weeks of daily 12-h noise exposures (42). Although this result is surprising, the relatively moderate level combined with the broadband frequency content of the noise exposures might explain the lack of vestibular loss observed in the Sohmer et al. (42) and Biron et al. (43) studies, compared to the high-level impulse noise used by Perez et al. (44), and the low-frequency high-level noise used by Stewart et al. (18, 45). Neurophysiological studies are consistent with anatomical studies that suggest the site, degree, and duration of damage observed in the vestibular periphery (temporary vs. permanent) is impacted by the level, frequency, and duration of noise exposure.

#### Cervical Vestibular Evoked Myogenic Potentials (cVEMPs)

**Figure 9:**
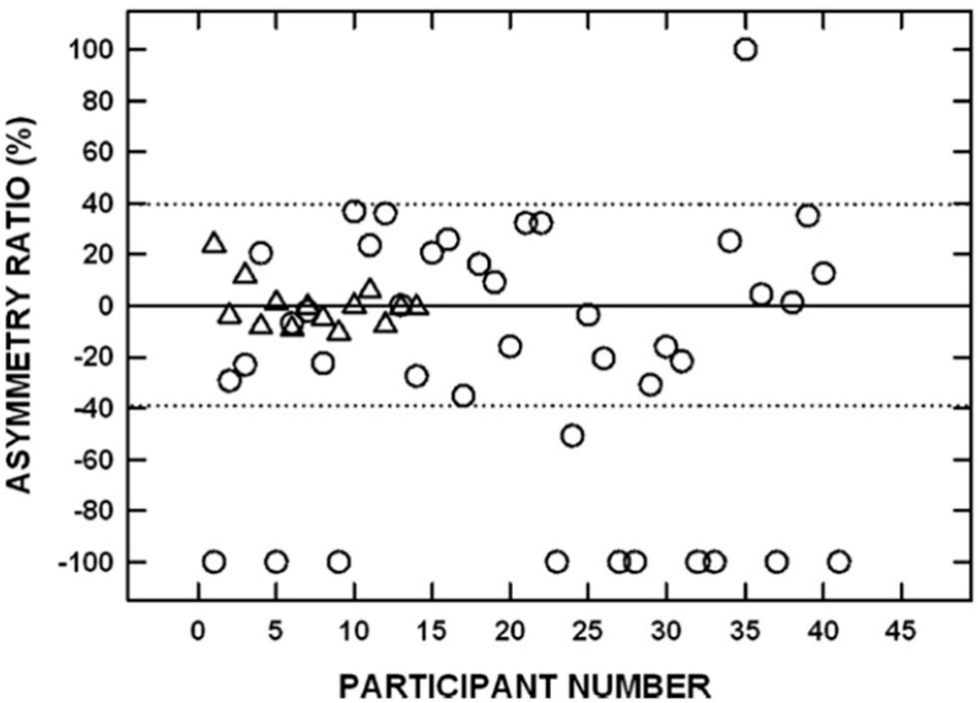
Signed interaural amplitude asymmetry ratio for cervical vestibular evoked myogenic potentials in 14 age-matched controls (triangles) and 41 participants with bilateral asymmetric noise-induced hearing loss (circles). For the noise-exposed group, −100% indicates the cVEMP was absent from the poorer-hearing ear, whereas 100% indicates the cVEMP was absent from the better-hearing ear. For the control group, negative values indicate that the P1-N1 amplitude was greater on the left side and positive values indicate that the P1-N1 amplitude was greater on the right side. The area between the dotted horizontal lines indicates asymmetry ratios within normal limits. Two noise-exposed participants had cVEMPs absent bilaterally and are not shown. Adapted from (53).

**Figure 10:**
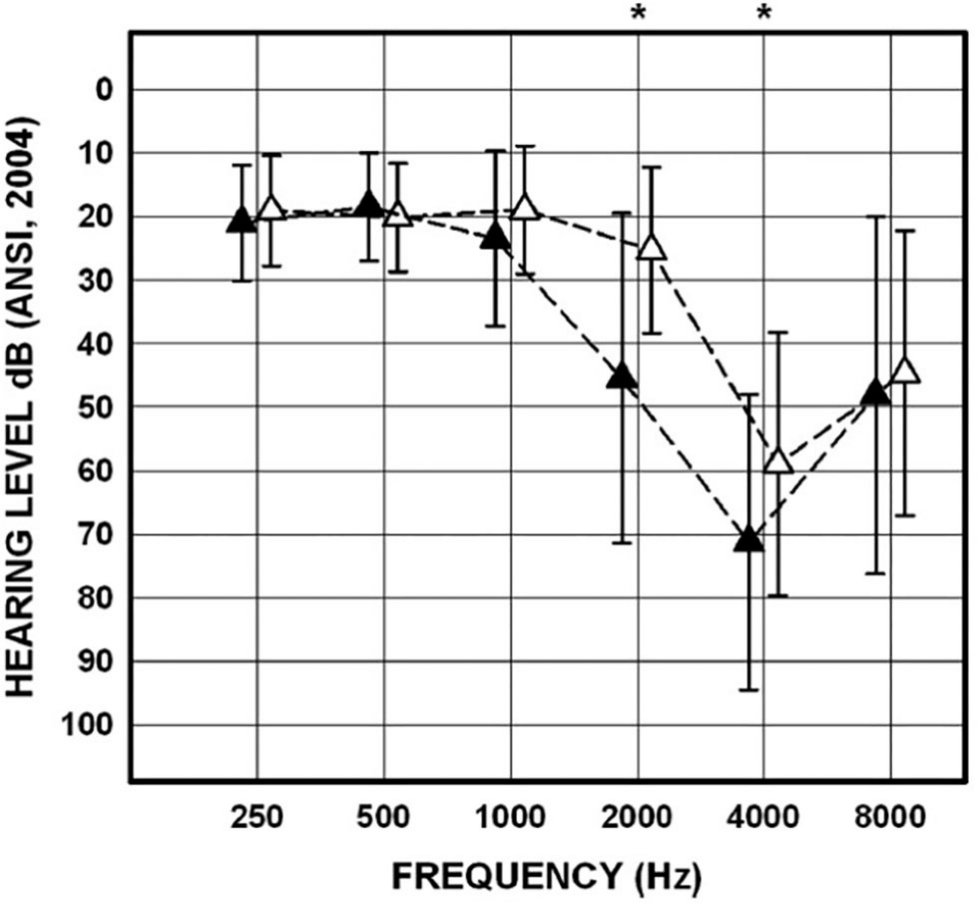
Mean and SDs for pure-tone thresholds for the poorer-hearing ear of noise-exposed participants (*n* = 43) with cVEMPs present (*n* = 29; open triangles) and for the poorer-hearing ear of noise-exposed participants with cVEMPs absent (*n* = 14; filled triangles). Asterisks indicate significant *post-hoc* comparisons. Adapted from (53).

The approach to determining noise-related damage to the human vestibular system has primarily focused on recording air-conducted sound cVEMPs in individuals with noise-induced hearing loss (NIHL). NIHL is characterized by an audiometric notch or “noise-notch” (decrease in hearing sensitivity at or near 4 kHz) and serves as a biomarker for noise-related damage to the cochlea. cVEMPs are absent in individuals with NIHL with an incidence ranging from 20 to 58% (53–60). Akin et al. (53) examined cVEMPs in 43 military veterans (mean age = 52 years) with a history of noise exposure greater in one ear than the other and asymmetric NIHL (defined as a noise notch at 4 kHz of ≥35 dB HL in the poorer hearing ear with a minimum interaural asymmetry of 20 dB HL at the affected frequencies). cVEMPs were absent in 24% of the poorer-hearing ears (Figure 9). In contrast, cVEMPs were present in most (97.5%) of the better-hearing ears of the noise-exposed group and present and symmetrical in the age-matched controls. Other studies have reported a decrease in cVEMP amplitudes and longer latencies in individuals with NIHL compared to individuals without noise exposure (54, 56, 59). Similarly, cVEMP thresholds were higher (poorer) in military veterans with NIHL than in age-matched controls (53). There is evidence that a diminished vestibular response is associated with the degree of NIHL. In military veterans with bilateral asymmetric NIHL, Akin et al. (53) observed that the poorer hearing ear of NIHL subjects with absent cVEMPs had a greater degree of high-frequency hearing loss than the poorer hearing ear of NIHL subjects with present cVEMPs (Figure 10). Similarly, in 30 industrial workers with NIHL, cVEMP latency increased and amplitude decreased as a function of a four-frequency pure-tone average (55). Wang et al. (57) examined hearing improvement following acoustic trauma in 20 patients and reported that absent cVEMP responses or abnormally prolonged cVEMP latency indicated poor prognosis for hearing recovery. Indeed, absent cVEMP or prolonged cVEMP latency predicted acoustic trauma hearing outcome with a sensitivity of 44% and specificity of 100%.

cVEMP findings in humans are consistent with morphological studies in animals that suggest the sacculus is particularly susceptible to noise-related damage. Human studies are limited, however, by a lack of histopathological data and difficulty quantifying noise exposure across a lifespan. Additionally, the human cVEMP is somewhat limited as an estimate of peripheral vestibular function as the response is recorded from the motoneurons of muscles at the end of a reflex pathway that includes central components. These limitations have been partially addressed by the work of Hsu et al. (16) in which cVEMPs were measured in guinea pigs following short-term vs. long-term noise exposure. In this study, a “normal” cVEMP was defined as the presence of a biphasic waveform at a latency of 6- to 9-ms, with a peak-to-peak amplitude of 5–20 μV. When a peak was not observed in the latency range of 6- to 9-ms, or was smaller than 5 μV, the cVEMP response was considered ‘abnormal'. Hsu et al. (16) observed recovery of vestibular function (return of normal cVEMP responses) following short-term (30 min) exposure to continuous broadband noise at 114 dB SPL. In contrast, abnormal cVEMP responses persisted for at least 30 days in most guinea pigs (78%; *n* = 18) following exposure to 40 h of continuous broadband noise. These findings are consistent with anatomical findings described earlier and suggest that permanent physiological damage to the sacculo-collic pathway is more likely following long vs. short-duration noise exposures.

#### Semicircular Canal Pathways

The studies that have examined the horizontal semicircular canal and VOR pathways have yielded inconsistent findings in individuals with NIHL. For example, Man et al. (61) observed a caloric weakness in only one of 176 patients with NIHL, whereas other studies have revealed a caloric weakness in up to 25% of individuals with NIHL (56, 57, 62). Using slow harmonic acceleration, Shupak et al. (63) found that VOR gain was significantly lower in industrial workers and military personnel with NIHL compared to a control group with normal hearing.

Recently, Yilmaz et al. (64) used the video head impulse test to measure VOR gain for all six semicircular canals in 36 industrial workers (mean age = 44 years) with high frequency hearing loss and four or more years working the steel and metal industry. They reported canal deficits (a decrease in VOR gain in at least one canal) in 55.5% of noise exposed participants compared with 6.6% of control participants. Decreased gain was reported more frequently in the horizontal semicircular canals (47%) than in the vertical canals (8%), with two noise-exposed participants demonstrating decreased VOR gain in more than one canal. Interpretation of these data is limited because NIHL was defined according to the degree of hearing loss at 4,000 Hz rather than a characteristic noise notch.

In contrast to cVEMP findings that suggest greater sacculo-collic pathway damage associated with more severe NIHL, the relationship between damage to the horizontal semicircular canal/VOR pathways and the degree of NIHL is less clear. Shupak et al. (63) observed significant correlations between pure-tone average, VOR gain, and caloric lateralization. Golz et al. (62), however, found no correlation between the severity of hearing loss and abnormal caloric test findings. It is worth noting, however, that the caloric response is a very low frequency response and may be independent of central zone hair cells and afferents that might be sensitive to pressure wave disturbances.

To examine the impact of noise exposure across multiple vestibular pathways, Tseng and Young (56) performed bithermal calorics and cVEMPs and oVEMPs using bone-conducted vibration on 30 individuals with NIHL related to chronic occupational noise exposure. Their findings revealed that cVEMPs were most frequently abnormal (70%) followed by oVEMPs (57%) and calorics (33%) consistent with other studies that suggest the saccule is more susceptible to acoustic trauma than other vestibular sensory organs. These findings are also consistent with anatomical findings that show the saccule is most susceptible to noise-induced damage, followed by the utricle, and then the semicircular canals (19).

VsEPs have also been used to assess semicircular canal function by replacing the linear head-jerk stimulus with an angular acceleration stimulus [A-VsEP; (42, 44)]. Perez et al. (44) delivered a 15,000°/s^2^ (1–3 ms rise time) stimulus to provoke A-VsEPs to assess semicircular canal function in sand rats (*Psammomys obesus*) exposed to impulse-noise (160 dB SPL, 10 impulses). This work found no change in A-VsEP amplitude, and only a transient (2–4-h) post-noise increase in A-VsEP latency that recovered by 1-week post-noise. In another study, the same stimulus was used to assess the effect of a short (1 h) or extended and repeated noise exposure (12 h per day for 21 days). There was no effect of noise exposure on the A-VsEP with either noise exposure paradigm (42). Furthermore, there was no effect of noise exposure on the linear VsEP following 113 dB SPL white noise exposure (42). It should be noted that there was a 7-day rest interval between the last 12-h noise exposure and the post-noise VsEP measurement. It is possible that if measurements had been taken shortly after the last noise exposure, a transient deficit might have been detected.

Single unit extracellular recording can be used to assess regular and irregular vestibular afferent activity arising from all five vestibular end organs. In a report characterizing changes in vestibular nerve activity, 116 dB SPL broadband white noise was delivered unilaterally to rats for 3 h on a single day. Seven days later, changes in hearing (ABR) and vestibular nerve activity (single unit extracellular recording from the superior vestibular nerve) on the noise exposed side was evaluated (19). Recordings from the superior aspect of the vestibular nerve included anterior and horizontal semicircular canal afferents as well as otolithic afferents (utricle and 1/3 of the saccule). Although there was no change in spontaneous firing rate in irregular superior vestibular nerve afferents, spontaneous firing rates were significantly reduced in regular superior vestibular nerve afferents originating from the anterior semicircular canal crista and the otolith organs. Furthermore, there were extensive changes in the gain and phase of regular horizontal and anterior canal afferents but a minimal effect of the 116 dB SPL broadband noise exposure on the irregular canal afferents. As discussed earlier, a post-exposure examination of vestibular sensory epithelia reflected broad damage to the hair bundles in all end organs innervated by the superior vestibular nerve (utricle, saccule; anterior and horizontal semicircular canal cristae). This work identified noise-induced damage to regular afferents and highlights a limitation of VsEP measurements: VsEPs only measure activity arising from irregular afferents.

### Central Vestibular Pathways

Using chronically implanted micro-electrode arrays, Ordek et al. (65) evaluated cerebellar neuronal activity after mild blast exposure (100–130 kPa). Behavioral testing 24 h and 7 days after blast (ladder climbing, roto-rod, ladder walking) and immunohistochemistry for the number of calbindin and caspase-3 (markers of Purkinje cells, oxidative stress, and apoptosis) positive cells showed no differences from controls. In contrast, evoked potentials after blast exposure demonstrated sustained changes beginning 24 h after injury. Potentials related to mossy fiber discharges exhibited increased amplitudes and latencies while potentials related to climbing fiber activity exhibited decreased amplitudes and decreased latencies. They concluded that neuronal activity may be more effective than behavioral tests or immunolabeling for neuronal loss in identifying early onset of subtle injury after mild blast exposure.

**Figure 11:**
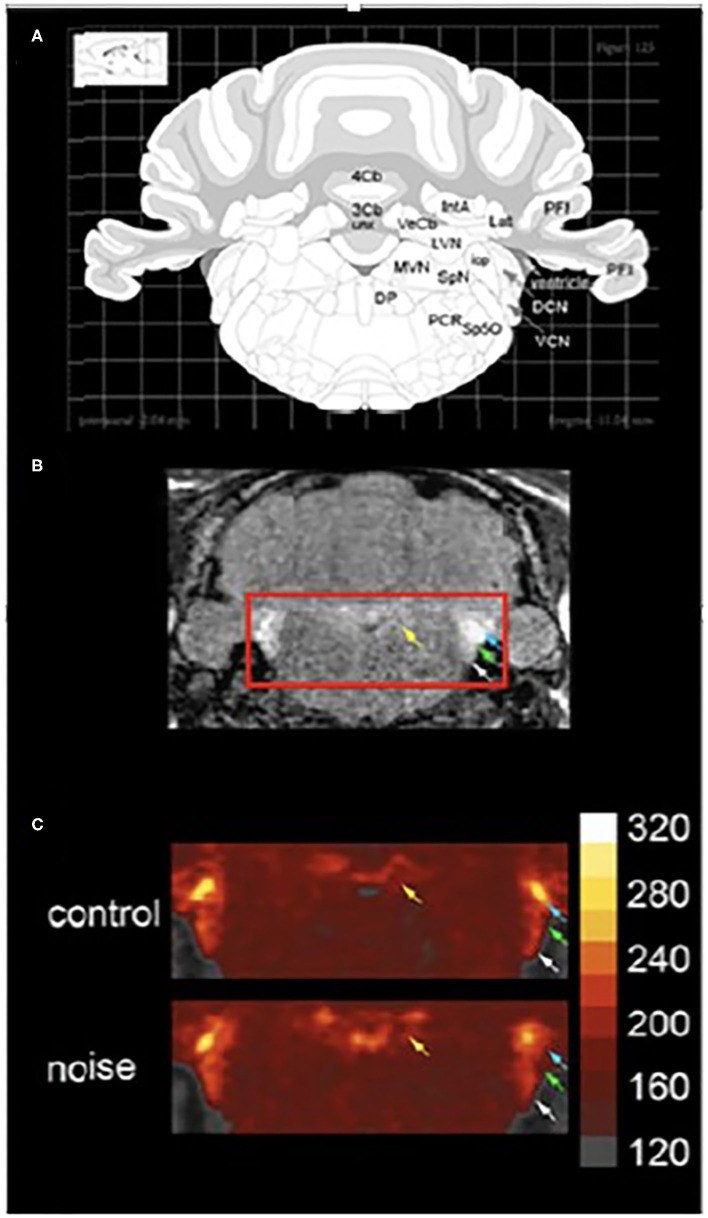
MEMRI (manganese enhanced magnetic resonance imaging) of the vestibular nuclear complex and cochlear nucleus 48 h. After noise exposure (10 kHz, 1/3 octave, 118 dB SPL, 4 h). **(A)** A schematic of a coronal section through the brainstem taken from a rat atlas showing brain regions of interest. **(B)** The T1-weighted image shows brain regions of interest *in vivo*. Yellow arrows indicate the vestibular nuclear complex while white, green, and blue arrows indicate auditory regions. **(C)** When noise-exposed animals are compared to controls 48 h. After the exposure there is increase in manganese uptake in the vestibular and auditory regions. Colorimetric scale bar–gray indicates lowest Mn2+ uptake while white indicates the highest level of Mn2+ uptake. Adapted from (68).

Other studies have made use of manganese enhanced MRI (MEMRI) to examine noise-induced changes in neuronal activity in animal models *in vivo* (66–68). Manganese is a paramagnetic ion that can be visualized using MRI. Manganese ions act as calcium ion surrogates and enters active neurons via voltage gated calcium channels. Differential uptake of manganese is used to identify changes in neuronal activity within vestibular-related brain regions at acute (48 h, Figure 11) and chronic time points (10 months, not shown) after noise exposure. Increased manganese uptake presumed to reflect increased neuronal activity, was found in the cerebellar paraflocculus and the primary visual cortex (68, 69). Although vestibular pathways were not a focus, this study provides a basis for future MEMRI studies that follow the impact of noise on temporal changes in neuronal activity within vestibular pathways *in vivo*.

## Functional Evidence for Noise-Induced Vestibular Damage

Loss of vestibular function can result in vertigo (the illusion of movement), oscillopsia (blurred vision during head movement), postural instability, and/or motion intolerance. Several studies in humans demonstrate a significant relationship between NIHL and postural stability. A limitation of these studies is a lack of vestibular function testing; thus, the mechanism underlying the association between NIHL and postural stability is not clear. An early study of iron workers (mean age = 53.3 years) with chronic noise exposure (70) demonstrated a significant relationship between auditory thresholds and postural stability as measured by sway velocity during static balance testing on a firm surface with eyes open and closed. Service members with NIHL (mean age = 44.6 years) due to chronic impulse noise exposure had greater postural sway (i.e., greater instability), especially in the medial-lateral direction, during static balance testing with eyes open and closed than controls with normal hearing and no noise exposure (mean age = 40.7 years) (71, 72). Guest et al. (73) showed a small reduction in voluntary limits of stability as measured by the functional reach test in military personnel with noise and solvent exposure (*n* = 601) compared to controls (no exposure: *n* = 391; noise exposure only, *n* = 500). Moreover, there was a significant inverse corelation between low-frequency auditory thresholds and functional reach, such that higher (poorer) thresholds were associated with lower functional reach.

Two studies failed to demonstrate a significant relationship between NIHL and postural stability (74, 75). Pyykkö et al. found equivalent postural sway velocity between Finnish soldiers (*n* = 54) with acute hearing loss due to firearm noise exposure (mean age = 27 years) and two control groups (soldiers with no acute noise trauma and age-matched civilians). Participants were tested within 5 days of the onset of hearing loss under a variety balance conditions: eyes open and closed on firm and foam surfaces, with and without calf muscle vibration, and with neck extended backwards. The lack of a significant relationship between NIHL and postural stability may be due to acute noise exposure (vs. chronic exposure in the previous studies) or the younger age of the subjects. Prasher et al. (75) examined postural control in four exposure groups: noise only (n = 153; mean age = 53.3 years), solvents only (n = 13; mean age = 49.6 years), noise and solvents (n = 174; mean age = 47.4 years) and controls with no noise or solvent exposure (n = 39; mean age = 47.6 years). As expected, the noise exposed group exhibited significantly poorer pure-tone thresholds than the other groups, but had normal postural stability as measured by computerized posturography during static balance with eyes open and closed on firm and foam surfaces. It is not clear why these findings conflict with the previous studies.

There is a paucity of data on the effects of noise exposure on agility and motor function in animal models. Tamura et al. (20) observed balance and gait changes in mice subjected to moderate level, low-frequency continuous noise for a one-month time period. Specifically, low-frequency noise-exposed mice exhibited impaired rotarod performance and imbalance, as well as shorter strides and a winding gait pattern that persisted for 4 weeks post-exposure vs. controls or high-frequency noise-exposed mice. These deficits were associated with reduced calbindin labeling of hair cells, and with elevated oxidative stress marker labeling when compared to control and high-frequency noise-exposed mice (20).

## Conclusions

This review has examined the current literature on noise-induced vestibular loss, the differences in characteristics of noise exposure, and how these differences might contribute to variability in reported noise-induced vestibular deficits. Early studies suggested that the vestibular system was susceptible to noise over-exposure. More recently, morphological studies have confirmed and extended this early work, showing cellular damage throughout the peripheral vestibular system, particularly in the otolith organs; however, there is still a paucity of data on the effect of noise exposure on human vestibular end organs. Other work has identified evidence of free-radical production in the vestibular labyrinth following noise exposure, which suggests a mechanism that may contribute to morphological observations. There are limited data on the effects of noise on the central vestibular system, especially following exposure to continuous noise. Physiological studies have corroborated morphological studies by demonstrating disruption across vestibular pathways with otolith-mediated pathways (VEMPs and linear VsEPs) impacted more frequently than semicircular canal-mediated pathways. Similar to the temporary threshold shifts observed in the auditory system, physiological studies in animals have suggested a capacity for recovery following noise-induced vestibular damage. Human studies have demonstrated that diminished VEMP responses are related to the severity of noise-induced hearing loss, and dose-dependent vestibular deficits following noise exposure have been corroborated in animal models. In contrast to the anatomical and neurophysiologic evidence, less is known about the relationship among noise-induced damage to the inner ear, physiological changes associated with this damage, and functional measures of vestibular impairment (e.g., balance and gait) in animals and humans.

## Author Contributions

CS reviewed the animal anatomical, physiological, and functional vestibular literature, drafted and revised the review. RA reviewed the anatomical literature and provided feedback on the entire review. AC drafted the blast-related hydrops section of the review and provided feedback on the entire review. CH drafted the human functional section of the review. AH drafted the central vestibular sections of the review and provided feedback on the entire review. OM reviewed physiological and functional literature, and revised the entire review. WK reviewed physiological literature and revised the entire review. FA reviewed the human anatomical, physiological, and functional literature, drafted and revised the review. All authors contributed to the article and approved the submitted version.

## Conflict of Interest

The authors declare that the research was conducted in the absence of any commercial or financial relationships that could be construed as a potential conflict of interest.
